# Soil Microbial Community Composition and Tolerance to Contaminants in an Urban Brownfield Site

**DOI:** 10.1007/s00248-022-02061-1

**Published:** 2022-07-08

**Authors:** Maura Palacios Mejia, Connie A. Rojas, Emily Curd, Mark A. Renshaw, Kiumars Edalati, Beverly Shih, Nitin Vincent, Meixi Lin, Peggy H. Nguyen, Robert Wayne, Kelsey Jessup, Sophie S. Parker

**Affiliations:** 1 Ecology & Evolutionary Biology, University of California, Los Angeles, Los Angeles, CA USA; 2 Ecology, Evolution, and Behavior Program, Michigan State University, Lansing, MI USA; 3 Natural Science, Landmark College, Putney, VT USA; 4 Cherokee Federal, USGS Wetland and Aquatic Research Center, Gainesville, FL USA; 5 Institute of the Environment and Sustainability, University of California, Los Angeles, Los Angeles, CA USA; 6 The Nature Conservancy, Los Angeles, CA USA

**Keywords:** Brownfields, Heavy metals, Contamination, Soil microbiome, eDNA, 16S rRNA, 18S rRNA, ITS

## Abstract

Brownfields are unused sites that contain hazardous substances due to previous commercial or industrial use. The sites are inhospitable for many organisms, but some fungi and microbes can tolerate and thrive in the nutrient-depleted and contaminated soils. However, few studies have characterized the impacts of long-term contamination on soil microbiome composition and diversity at brownfields. This study focuses on an urban brownfield—a former rail yard in Los Angeles that is contaminated with heavy metals, volatile organic compounds, and petroleum-derived pollutants. We anticipate that heavy metals and organic pollutants will shape soil microbiome diversity and that several candidate fungi and bacteria will be tolerant to the contaminants. We sequence three gene markers (16S ribosomal RNA, 18S ribosomal RNA, and the fungal internal transcribed spacer (FITS)) in 55 soil samples collected at five depths to (1) profile the composition of the soil microbiome across depths; (2) determine the extent to which hazardous chemicals predict microbiome variation; and (3) identify microbial taxonomic groups that may metabolize these contaminants. Detected contaminants in the samples included heavy metals, petroleum hydrocarbons, polycyclic aromatic hydrocarbons, and volatile organic compounds. Bacterial, eukaryotic, and fungal communities all varied with depth and with concentrations of arsenic, chromium, cobalt, and lead. 18S rRNA microbiome richness and fungal richness were positively correlated with lead and cobalt levels, respectively. Furthermore, bacterial *Paenibacillus* and *Iamia*, eukaryotic *Actinochloris*, and fungal *Alternaria* were enriched in contaminated soils compared to uncontaminated soils and represent taxa of interest for future bioremediation research. Based on our results, we recommend incorporating DNA-based multi-marker microbial community profiling at multiple sites and depths in brownfield site assessment standard methods and restoration.

**Supplementary Information:**

The online version contains supplementary material available at 10.1007/s00248-022-02061-1.

## Introduction

About 15% of urban land parcels in the USA are brownfields—abandoned industrial or commercial facilities that can harbor hazardous contaminants, which constrain their development and limit their use due to risks posed to humans and wildlife [1]. In addition, because of the industrial activities, the soils found in brownfields are often nutrient-depleted, highly saline or alkalinized, and highly compact, affecting plant, animal, and microbial communities [2–7]. The reduced nutrients in the soil of brownfields, particularly of C, N, P, Na, and K, can directly limit enzymatic activity and microbial biomass [7, 8]. Persistent organic contaminants at these sites can include polycyclic aromatic hydrocarbons (PAHs), polychlorinated biphenyls (PCBs), and brominated flame retardants (BFRs) [9–11], which reduce the availability of organic matter in soils, and in turn reduce microbial diversity [12]. Benzene which creates anoxic conditions and reduces redox potentials during oxidation and trichloroethene (TCE) which affects microbial preferential C substrate uptake may also be present. Inorganic pollutants such as the heavy metals lead, cadmium, and mercury are also common in brownfield soils and toxic to many bacteria and eukaryotes [13, 14]. Some heavy metals (e.g., zinc, cobalt, and manganese) are essential for bacterial survival and functioning but become harmful at high concentrations [15]. Overall, heavy-metal, PAH, benzene, and TCE contamination exert selective pressures on soil microbial communities and can over time reduce microbial diversity by eliminating sensitive taxa and disrupting soil health and function [16].

Nonetheless, these same selective pressures can also favor the growth of taxa that are tolerant or resistant to the pollutants and may be involved in contaminant biodegradation or bioaccumulation [17]. A variety of bacteria and fungi have mechanisms that transform, neutralize, accumulate, or excrete pollutants [18–20]. Thus, brownfield sites can sustain diverse, active ecological communities despite the hazardous soil characteristics. Microbial group assemblages capable of biodegrading petroleum hydrocarbons in brownfield sites include *Acinetobacter*, *Brevibacterium*, *Brevundimonas*, *Corynebacterium*, *Microbacterium*, and *Stenotrophomonas* [21]. *Micrococcus* sp. has been found to be tolerant and potentially resistant to chromium and nickel in industrial wastewaters [22, 23]. The abundances of *Rhodoferax ferrireducens* and *Dechloromonas aromatica* increase in groundwater contaminated with benzene [24]. Several bacterial groups (*Desulfuromonas*, *Pelobacter*, *Desulfovibrio*, and *Fusibacter*) increase in abundance in surface sediments exposed to PCE and TCE [25]. Furthermore, filamentous fungi isolated from the genera *Aspergillus*, *Penicillium*, and *Fusarium* show tolerance to various heavy metals (Zn, Pb, Cr, Cd, Ni, and Co) in vitro, and *Alternaria* specifically has been isolated for its PAH-degrading capabilities [26, 27]. The abundances of Cryptomycota, Dothideomycetes, *Preussia*, and *Corynespora* increase in the presence of heavy metals or PAHs in contaminated soils, suggesting that these might be bioindicators of contamination [28].

**Fig. 1:**
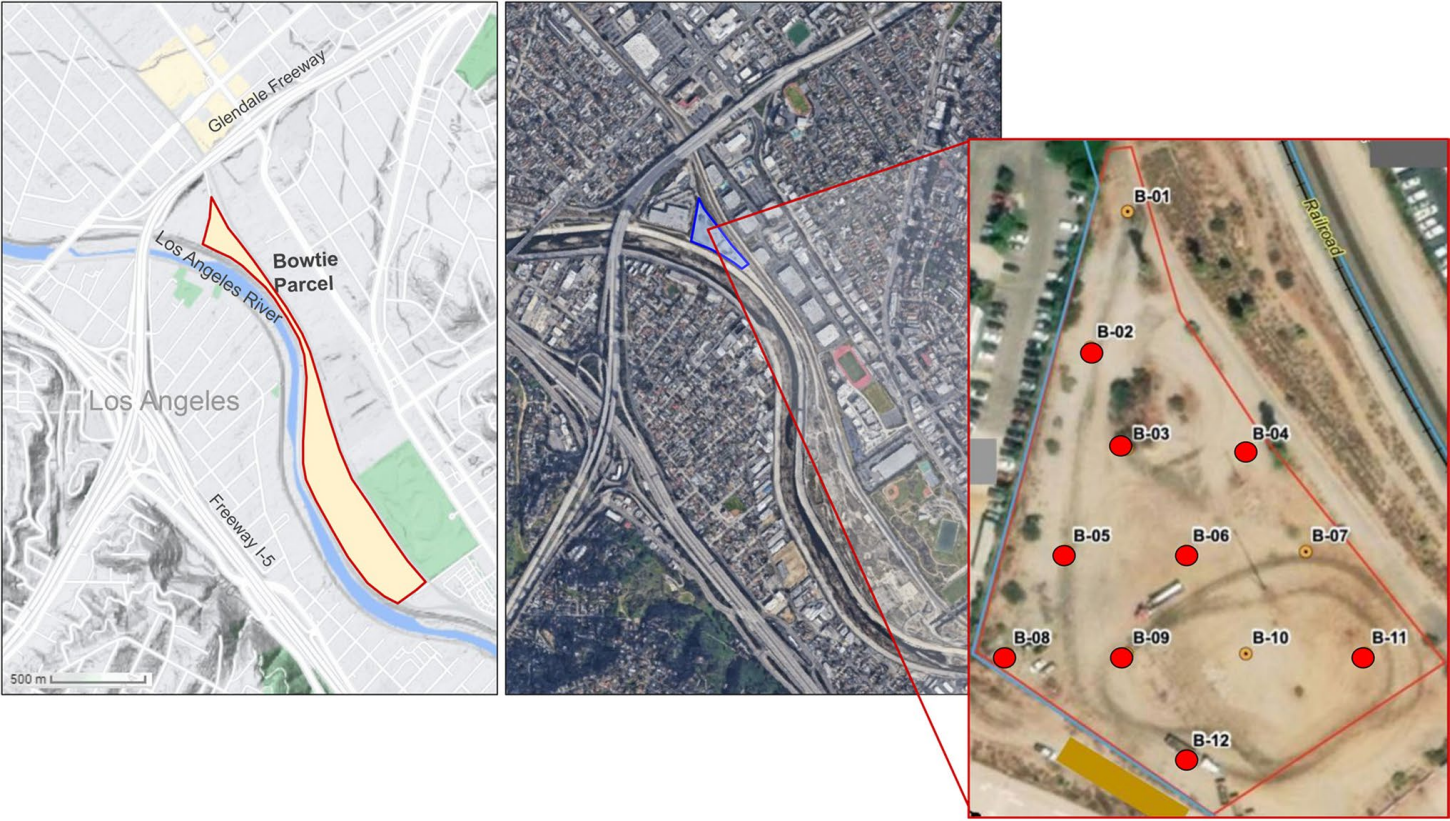
Study site and sampling design. Aerial view of the sampling location, known as the “Bowtie” property within Taylor Yard—a former rail yard in Los Angeles, CA, that is owned and managed by California State Parks. Soil samples were taken from below ground (at 0.1524 m, 1.524 m, 3.048 m, 4.572 m, and 6.096 m) at 12 ordinary sites, labeled B-01 to B-12. Samples from the surface (0.15 m) were collected by hand, and all other samples were collected using a direct-push drill rig in an acetate sleeve. Sampling locations with a red dot signify contaminants present at the surface as contamination at excess levels was only found at the surface

Despite the growing research on the microbial ecology of brownfields, few studies have focused on soil microbiome responses to long-term contamination across multiple depths in brownfield sites, which is necessary for capturing contamination that may have leached from the surface to deeper layers. Furthermore, baseline surveys of the soil microbiome in brownfields can aid in assessing the levels of biodiversity, nutrient cycling, and ecosystem services supported at these sites, and inform restoration plans. Here, we use eDNA metabarcoding to characterize the soil microbiome across varying depths at a former rail yard that ceased operations in the late 1980s (Fig. 1). The study site is contaminated with heavy metals and organic pollutants including total petroleum hydrocarbons (TPHs) and polycyclic aromatic hydrocarbons (PAHs). Specifically, our study objectives were to (1) characterize bacterial, eukaryotic, and fungal soil microbiome composition and diversity using 16S rRNA, 18S rRNA, and the fungal internal transcribed spacer (FITS) markers; (2) determine whether hazardous substances are correlated with soil microbiome variation; and (3) highlight the microbial taxa that are enriched in contaminated soils to assist in the identification of potential candidates for bioremediation. We anticipate that heavy metals and organic pollutants will shape soil microbiome alpha and beta diversity, and that certain fungi and bacteria will be enriched in contaminated soils and potentially tolerant to contamination as previously documented for other brownfield sites. Collectively, our study will provide a greater understanding of soil microbiome resiliency and the impacts of environmental pollutants on the soil communities at an urban brownfield site, which can inform future restoration efforts.

## Materials and Methods

### Study Site

This study was conducted at the “Bowtie property” located within Taylor Yard, a former rail yard in Los Angeles that was operated by the Union Pacific Railroad since the 1920s (Fig. 1) [29, 30]. The railyard was used for railroad car engine maintenance and fueling, and pollutants that were commonly used at the site included diesel fuel, lubrication oils, gasoline, acids, cleaning solvents, alkaline soaps, paint and thinners, lead, pesticides, and herbicides [29, 30]. Operations ceased in 1985 and in 2003, the land was purchased by California State Parks.

The study site is adjacent to the channelized, natural soft-bottom Los Angeles River and is surrounded by a mix of residential and industrial facilities (Fig. 1). The soil at this site is hydrophobic, compact, and nutrient-depleted, thus, much of the property remains barren apart from small patches of weeds [30]. The soil consists of gravel, asphalt, and sand layered with concentrations of silt and clay from the surrounding hillsides. Currently, detected pollutants in the surface soils of Taylor Yard include heavy metals (lead, arsenic, cadmium, cobalt, mercury, and vanadium), benzene, chlorinated solvents (PCE), and petroleum hydrocarbons [30]. The City of Los Angeles plans to redevelop the former rail yard as a public river park with vibrant greenspace that will bring back native wildlife and plants.

### Sample Collection and Pollutant Quantification

**Fig. 2:**
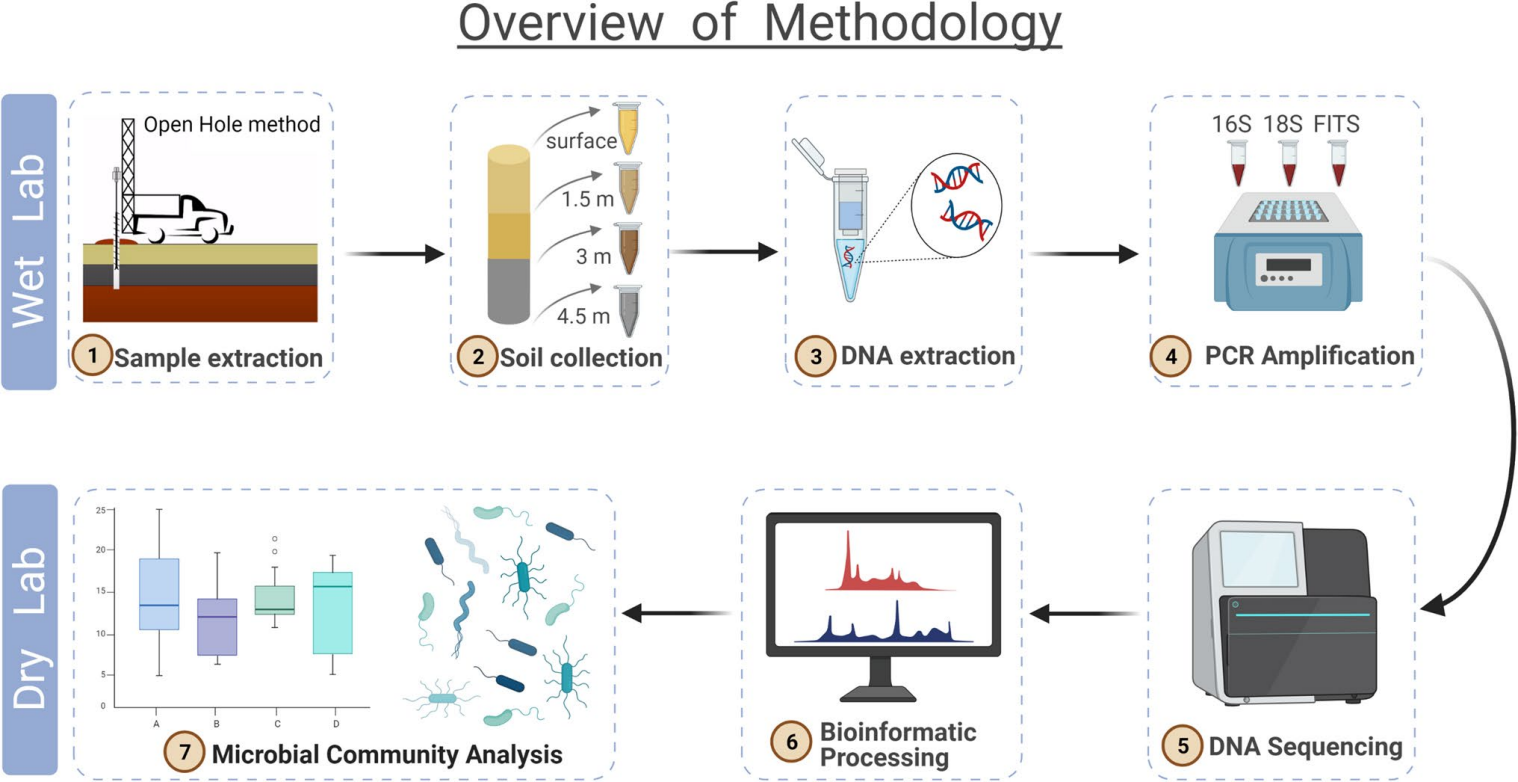
Overview of the laboratory and computational workflow of this study. Briefly, soil samples were collected from five depths at the “Bowtie” site within Taylor Yard, a former rail yard in Los Angeles, CA, that is now owned and managed by California State Parks. After DNA extraction, two rounds of PCR amplified three targeted metabarcodes for microbes (16S rRNA marker, 18S rRNA marker, and fungal ITS marker). Sequences were processed in Anacapa and DADA2, generating a table of amplicon sequence variants counts for each sample. This ASV table along with sample metadata was uploaded into the R statistical program for statistical analyses and visualizations

Soil samples (*N* = 55) were collected from 5 distinct depths (0.1524 m, 1.524 m, 3.048 m, 4.572 m, and 6.096 m) at 12 sites within two acres of the location (Fig. 1) (Table S1). Three samples at 0.15 m were also collected from an uncontaminated neighboring site to serve as controls (Table S1); subsurface samples were not collected due to permit requirements. Soil was insufficient sampled for two samples and a buried pipe inhibited the collection of three samples from site 9. Therefore, the final sample sizes for each depth were as follows: 0.15 m (*N* = 12), 1.52 m (*N* = 11), 3.04 m (*N* = 11), 4.06 m (*N* = 10), and 6.09 m (*N* = 11). Disposable scoops or a hardened steel-core barrel lined with an acetate sleeve were used to collect samples. The 55 samples were subsampled three times to obtain triplicate samples, and these were stored in 15-oz sterile glass containers at 4 °C. Samples were transported to the University of California, Los Angeles for DNA extraction (Fig. 2). Sampling methods are described in further detail in the supplementary materials.

Of the 55 soil samples collected, only samples from three depths (0.15 m, 1.52 m, and 6.09 m) were screened for pollutants to quantify the penetration of the contaminants from the surface to the deeper areas (*N* = 34, Table S2). Specifically, they were processed for total petroleum hydrocarbons as diesel (TPH-d), total petroleum hydrocarbons as motor oil (TPH-mo) [31], 17 California Assessment Manual (CAM 17) heavy metals, and 16 polycyclic aromatic hydrocarbons (PAHs) (Table S2). Samples from the other surveyed depths were not processed for pollutants to reduce costs associated with the project.

### Sample Processing: Environmental Parameters and eDNA Metabarcoding Library Preparation

Triplicate soil samples, subsampled from each of our 55 original soil samples, were taken to reduce the effect of random variation and to capture a representative sample of the microbial community at our site. These were then pooled for DNA extractions using QIAGEN DNeasy PowerSoil kits, along with three extraction blanks (Fig. 2). Two rounds of polymerase chain reaction (PCR) were used to amplify three targeted metabarcodes for microorganisms from the Earth Microbiome Project: 16S rRNA [32], 18S rRNA [33], and fungal ITS (FITS) [34, 35]. The three-marker approach was used to reduce primer bias and obtain more robust conclusions (De Gruyter et al. 2020). Detailed PCR instructions can be found in the Supplementary Materials.

The DNA concentration was quantified with the Qubit dsDNA HS Assay (Life Technologies) so libraries could be pooled to equal molar concentrations for Illumina MiSeq sequencing (Fig. 2). The sequencing was conducted at the Genomics and Bioinformatics center in Notre Dame University (IN, USA), using the MiSeq Reagent Kit v3 (600-cycle, 2X300-bp paired end). A single PCR negative control was included, using sterile water in place of template DNA.

### Sequence Processing

Raw sequences were processed using the Anacapa QC toolkit [36] (Fig. 1). Briefly, reads were quality-controlled, sorted, and assigned into amplicon sequence variants (ASVs) using the Divisive Amplicon Denoising Algorithm (DADA2) module embedded in Anacapa [36]. The DADA2 pipeline filters, dereplicates, identifies chimeras, and merges paired end reads [37]. The Anacapa classifier was used to assign taxonomic origins for ASVs that include forward, reverse, and merged reads from compiled GenBank databases [38]. Taxonomic assignment tables were used with a bootstrap confidence cutoff of 60.

The R [39] decontam package v. 3.4.2 [40] was used to remove contaminant ASVs for each metabarcode marker (16S rRNA, 18S rRNA, and FITS) and sequencing run, based on their prevalence in extraction blank control samples. ASVs with decontamination scores below the specified threshold (0.1) or not assigned a taxonomic path were removed from the dataset unless they had the same taxonomic classification as an uncontaminated ASV. After sequence processing, samples had an average of 145,789 sequences for 16S rRNA data, 78,568 sequences for 18S rRNA data, and 1971 sequences for FITS data. Due to their low number of reads (< 200 sequences), a total of 35 out of 55 samples were removed from the FITS dataset and were excluded from all subsequent analyses.

### Assessing Microbial Community Composition

Unless otherwise stated, all statistical analyses and figures were generated using the statistical software package R (v. 3.6) [39]. Community composition for each marker (16S rRNA, 18S rRNA, and FITS) was visualized through stacked bar plots using the ggplot2 package [41]. The plots showed the relative abundances of dominant phyla and orders to compare community composition across depths. Microbial abundance data was not rarefied for these plots, but identical plots using rarefied data are provided as supplementary figures.

### Alpha Diversity Analyses

Prior to assessing alpha diversity, samples were rarefied to 42,000 reads per sample (for 16S rRNA data), 21,000 reads per sample (for 18S rRNA data), and 370 reads per sample (for FITS data) using mothur [42, 43]. These cutoffs represented the third lowest number of sequences in each dataset; selecting the lowest number of sequences as the cutoff would have been too restrictive. A total of 4 different samples did not meet this sequencing cutoff for 16S rRNA, 18S rRNA, and FITS data, and were excluded from alpha diversity analyses. Rarefaction curves of ASV richness reached saturation, indicating that sequencing depth was sufficient for analyzing these communities (Fig S1).

Community alpha diversity was estimated using Chao 2 richness from ASV presence/absence (e.g., incidence) data using the fossil package [44]. To identify significant drivers of soil microbiome alpha diversity, we constructed three linear mixed models using the lme4 package [45]. All three models set soil site (e.g., soil core) as a random factor and Chao 2 richness (on the log scale) as the independent variable. Log transformation is typically recommended for data that is spread out or when the relationship between x and y is not linear [46]. The transformation helps with model linearity, which is an important mathematical assumption of the regression model, and it overall improves the accuracy of the model [46].

Model 1 included sample depth (m) as a predictor. Model 2 only analyzed data from the surface, including data from the uncontaminated neighbor lot, and related Chao 2 richness (log) with a binary category for contamination (contaminated vs. uncontaminated). The category was based on whether the concentrations of heavy metals or hydrocarbons in the sample exceeded regional screening guidelines (see Supplementary Materials for more information). Model 3 was restricted to samples with known concentrations of hydrocarbons and heavy metals, and specified depth, and concentrations of arsenic, cobalt, chromium, lead, and benzo(a)pyrene (all on log scale) as predictors of community diversity (see Supplementary Materials and Table S2 for how these particular pollutants were selected). After assessing model fit using residuals, likelihood ratio tests (LRT) were conducted on these models using the R car package [47] to identify the significant predictors.

### Beta Diversity Analyses

Microbial beta diversity was estimated using Jaccard distances calculated from unrarefied bacterial ASV presence/absence data using the vegan package [48]. Permutational multivariate analysis of variance (PERMANOVA) tests with 999 permutations were used to identify significant predictors of soil microbial community structure. PERMANOVA models identical to the alpha diversity models were constructed to identify predictors of soil microbiome variation. All three PERMANOVA models set the soil site as strata. The clustering of ecological communities for each marker was visualized via principal coordinate analysis (PCoA) plots constructed from Jaccard distance matrices using ggplot2. To further evaluate whether heavy-metal or hydrocarbon concentrations (Table S2-S3) were correlated with soil microbiome structure, constrained correspondence analysis (CCA) was conducted using the cca function from the vegan package. Results from CCA analyses were visualized using ggplot2.

Lastly, to identify microbial groups that may be tolerant to contamination, we ran linear discriminant analysis effect size (LEfSe) tests with default parameters [49]. The samples analyzed were from the surface and were categorized as “contaminated” or “uncontaminated,” similarly to what was done for alpha and beta diversity analyses. The category was based on whether the concentrations of heavy metals or hydrocarbons in the sample surpassed regional screening levels. Unrarefied data was used and only microbial genera > 0.01% average relative abundance across these samples were included in the data frames for each marker. When the microbial genus was unknown, the last known classification was used (e.g., family). Output from LEfSe of statistically significant microbial “genera” (LDA > 3) was visualized via diverging plots with the ggplot2 package. Similar methods were followed to identify microbial taxa that were differentially abundant among depths (0.15–1.5 m vs. 3.05–4.60 m vs. 6 m). For this, bacterial, eukaryotic, and fungal communities were collapsed at the level of phylum, order, or genera. Only microbial groups with > 0.01% average relative abundance across samples were retained for upload to LEfSe.

## Results

### Composition of the Soil Microbiome

**Fig. 3:**
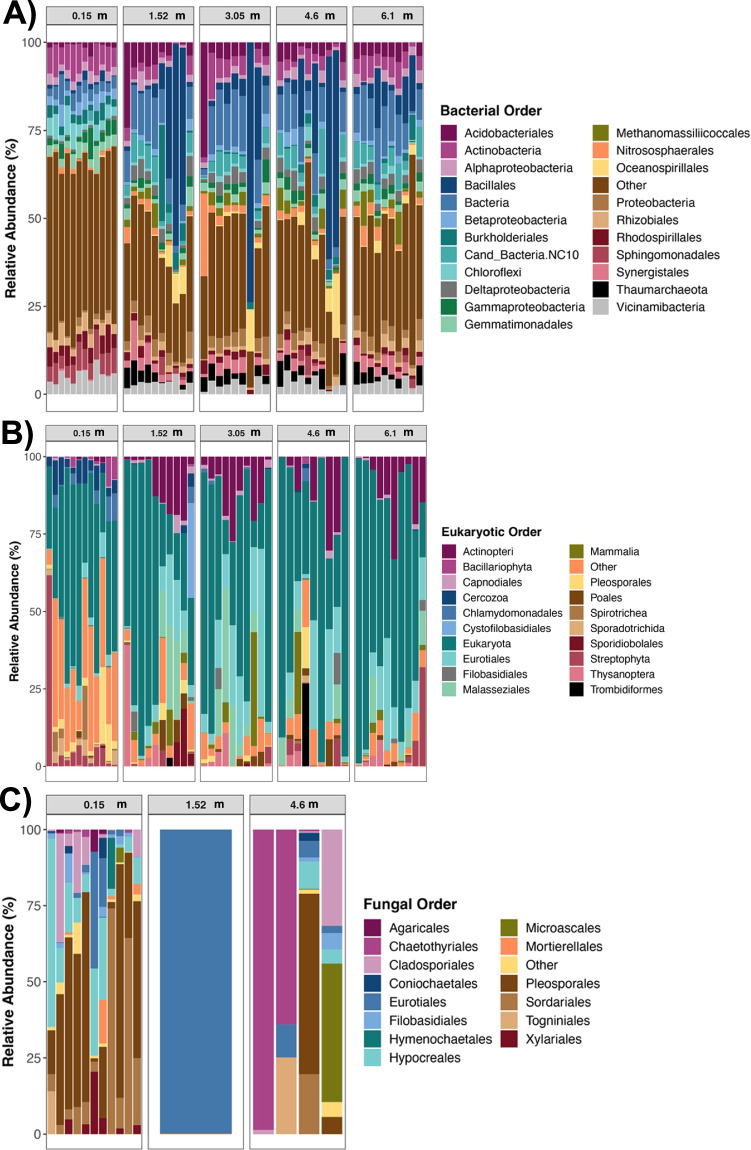
Community composition of the soil microbiome in a contaminated brownfield site. Stacked bar plots showing the relative frequency of sequences assigned to each microbial order across samples for **A** 16S rRNA profiles, **B** 18S rRNA profiles, and **C** fungal ITS profiles. Samples are grouped by depth (m), and each color represents a microbial order. Microbial abundance data was rarefied for these plots

We characterized the composition of soil microbial communities across five depths in a site contaminated with heavy metals, petroleum hydrocarbons, and PAHs. At the phylum level, the 16S rRNA soil microbiome profiles were dominated by Proteobacteria (28% mean relative abundance), Firmicutes (15%), Actinobacteria (12%), and Acidobacteria (9%) (Fig S2). The Proteobacteria mostly consisted of Alpha (8.3%), Gamma (6.5%), Beta (4.82%), and Deltaproteobacteria (4.83%) (Fig. 3). 18S rRNA microbiome profiles were mostly composed of unclassified Eukaryota (54% mean relative abundance), Ascomycota (12%), Chordata (9%), Basidiomycota (7.4%), and Streptophyta (4.91%) (Fig S2). Fungal communities were dominated by a single phylum, Ascomycota (94%) (Fig S2), specifically by the Dothideomycetes (37.7%), Sordariomycetes (34.9%), and Eurotiomycetes (21.8%) classes. For the pollutant quantities at each depth, see Table S2.

For the three markers, soil microbiome composition appeared to vary with depth (Fig. 3). Statistically, LEfSe analyses identified several taxa at the phylum, order, and genera level as being differentially abundant among depths (Table S4). For bacterial profiles, samples from 0.5 to 1.5 m harbored greater abundances of *Longimicrobium* and *Thermoleophilum* compared to the other depths, while the deepest samples (6 m) had relatively more *Pedomicrobium* (LEfSe LDA > 3, *p* < 0.05; Table S4). No phyla were differentially abundant among the different depths for 16S rRNA profiles. For eukaryotic profiles, the abundances of only two phyla—Cryptophyta and Ciliophora were distinct among depths; specifically, they were enriched in samples from 0.5 to 1.5 m relative to samples from the other depths (LEfSe LDA > 4, *p* < 0.05; Table S4). None of the orders or genera varied with depth for this dataset according to LEfSe analyses. Lastly, for fungal communities, several taxa including Sordariales, Hypocreales, and Pleosporales, were particularly abundant at 0.5–1.5 m than at other depths. *Preussia* were more abundant in samples from deeper depths compared to surface samples (LEfSe LDA > 4.5, *p* = 0.03; Table S4).

### Correlates of Soil Microbiome Alpha Diversity

Soil microbiome Chao 2 richness decreased with depth for 18S rRNA and fungal ITS profiles (LMM LRT *p* < 0.05; Table S5). No significant relationship was observed between microbiome richness and sample depth for the 16S rRNA data (LMM LRT *p* > 0.05; Table S5). Furthermore, microbiome richness did not differ between contaminated and uncontaminated surface samples for any of the three markers analyzed (LMM LRT *p* > 0.05; Table S5). Lastly, while the microbiome alpha diversity of 16S rRNA data did not significantly correlate with concentrations of heavy metals or hydrocarbons, those of 18S rRNA and fungal ITS profiles did (LMM LRT *p* < 0.05; Table S5). Specifically, 18S rRNA community richness was positively correlated with lead concentrations (1.6–140 mg/kg) while fungal community richness was positively associated with cobalt concentrations (3.5–11 mg/kg) (Table S5, Fig S3). See the Supplementary Materials for a more in-depth report of the levels of contamination found across our study samples.

### Correlates of Soil Microbiome Beta Diversity

Similar to alpha diversity analyses, beta diversity analyses evaluated whether soil depth and degree of contamination accounted for variation in soil microbiome profiles, after accounting for variation among soil sites. Soil microbial communities were partitioned by depth, and this variable explained 6–9% of the variation in 16S rRNA, 18S rRNA, and FITS profiles (PERMANOVA *p* < 0.05; Table 1). Furthermore, surface samples from sites with elevated levels of heavy metals and hydrocarbons (i.e., where contaminant concentrations exceeded regional screening guidelines) did not harbor significantly different soil communities than surface samples from uncontaminated sites (PERMANOVA *p* > 0.05; Table 2). Nonetheless, in a PCoA ordination contaminated samples do cluster separately from uncontaminated sites, particularly from samples of the neighboring lot (Fig S4).

**Table 1:** Soil microbiome structure varies with sampling depth

Marker	Model parameter	% variance explained	*p* value
16S rRNA	Depth	**5.91**	**0.001**
18S rRNA	Depth	**7.66**	**0.001**
FITS	Depth	**9.06**	**0.052**

Shown here are the *R*.^2^ values (% variance explained) and *p* values for PERMANOVA models that tested the influence of sample depth and included soil site as strata. The PERMANOVA tests were based on Jaccard distance matrices and all samples were included in the analysis except samples from the uncontaminated neighboring lot. Results for each marker are shown above. Statistically significant values (*α* = 0.05) are bolded

**Table 2:** Soil microbiomes are not distinct between contaminated and non-contaminated surface soils

Marker	Model parameter	% variance explained	*p* value
16S rRNA	Contaminated vs. uncontaminated soils	8.6	0.9
18S rRNA	Contaminated vs. uncontaminated soils	9.26	0.9
FITS	Contaminated vs. uncontaminated soils	15.71	0.9

Shown here are the *R*.^2^ values (% variance explained) and *p* values for PERMANOVA models that tested the influence of contamination (yes vs. no) in surface soils on microbiome structure, while setting soil location as strata. The PERMANOVA tests were based on Jaccard distance matrices and only samples from the surface were included in the analyses. Surface samples from an uncontaminated neighboring lot were also included. Results for each marker are shown above

**Fig. 4:**
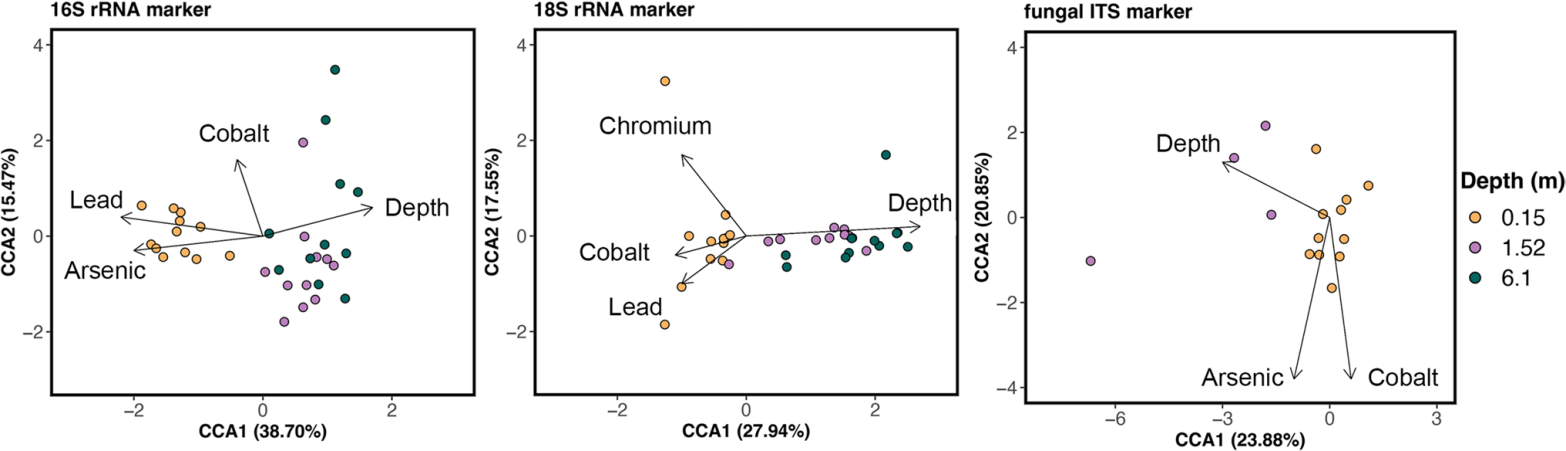
Soil microbiome structure varies with concentrations of heavy metals and hydrocarbons in a brownfield site. Canonical correspondence analysis (CCA) correlated soil microbiome structure at the sampled depths with concentrations of arsenic, cobalt, chromium, lead, and benzo(a)pyrene. Only samples from depths with known concentrations of contaminants (0.15 m, 1.52 m, and 6.10 m) are included. The two primary CCA axes are shown and samples are color-coded by depth for each marker. Arrows indicate the direction and magnitude of statistically significant relationships (at *α* = 0.05). See Table S6 for the detailed statistical output

Lastly, of the 6 heavy metals and hydrocarbons included in our statistical models, concentrations of cobalt (3.5–11 mg/kg) and lead (1.6–140 mg/kg) significantly predicted microbiome structure for 16S and 18S rRNA profiles, even after accounting for variation attributable to sample depth (PERMANOVA *p* < 0.05; Table 3). 16S rRNA profiles were also correlated with concentrations of arsenic (1–4.3 mg/kg) and chromium (8–110 mg/kg; Table 3). Fungal community structure was not significantly associated with any of the heavy metals analyzed (PERMANOVA *p* > 0.05; Table 3). These findings were consistent with the output of constrained correspondence analysis (CCA) with a few exceptions (Fig. 4, Table S6): 16S rRNA communities were not strongly associated with chromium concentrations but 18S rRNA profiles were strongly associated with this metal. Fungal communities were significantly predicted by cobalt and arsenic concentrations in this analysis (Fig. 4, Table S6).

**Table 3:** Concentrations of heavy metals and hydrocarbons predict soil microbiome structure

Marker	Statistic	Arsenic	Cobalt	Chromium	Lead	Benzo(a)pyrene	Depth
16S rRNA	% variance explained (*p* value)	**4.05 (0.032)**	**6.38 (0.01)**	**4.56 (0.001)**	**5.43 (0.001)**	2.12 (0.85)	4.17 (0.12)
18S rRNA	% variance explained (*p* value)	2.98 (0.26)	**7.51 (0.001)**	3.7 (0.12)	**5.5 (0.001)**	2.1 (0.93)	**4.82 (0.027)**
FITS	% variance explained	9.47 (0.12)	6.24 (0.56)	5.07 (0.12)	7.88 (0.56)	5.15 (0.43)	14.54 (0.062)

Shown here are the *R*.^2^ values (% variance explained) and *p* values for PERMANOVA models that determined whether five soil contaminants were associated with variation in soil microbiomes. The PERMANOVA tests were based on Jaccard distance matrices and only samples screened for soil analytes were included in the analyses. Soil location was set as strata and depth was also included as a predictor in the model. Results for each marker are shown above. Statistically significant values (*α* = 0.05) are bolded

### Heavy-Metal Tolerant Microbial Taxa Enriched in Contaminated Soils Relative to Uncontaminated Soils

**Fig. 5:**
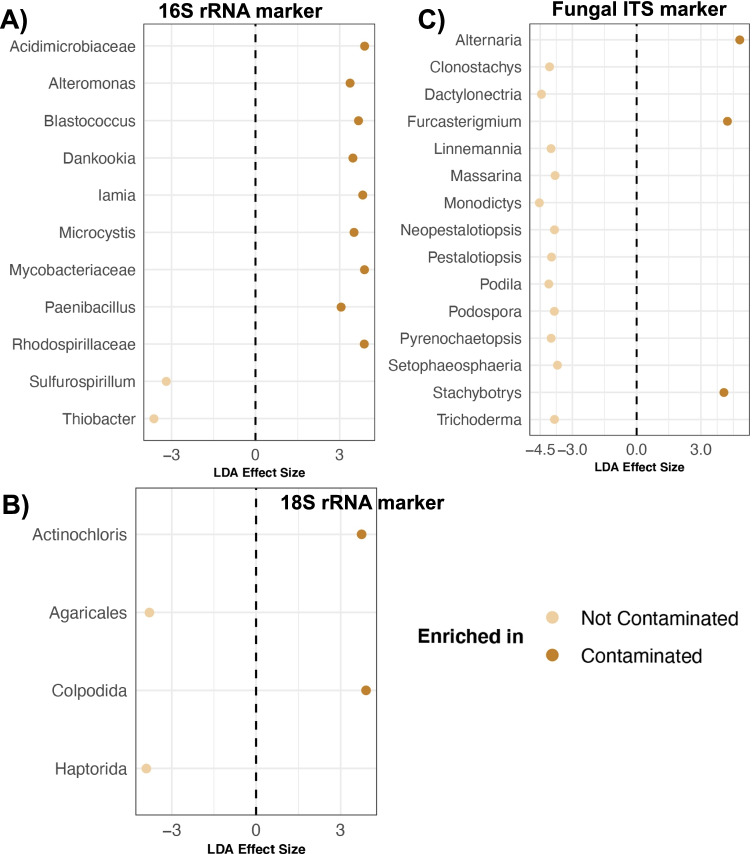
Microbial groups associated with contaminated surface soils represent potential sources for bioremediation efforts. Taxa enriched in contaminated vs. uncontaminated surface samples as determined by LEfSe for **A** 16S rRNA, **B** 18S rRNA, and **C** fungal ITS community profiles. Each dot represents a unique group and is color-coded by soil contamination (yes vs. no). Statistically significant genera (LDA > 3) are displayed and their most specific taxonomic classifications are on the x-axis. See methods for more details regarding the analysis

We compared the abundances of microbial taxa in contaminated vs. uncontaminated surface soils using linear discriminant analysis effect size (LEfSe) to identify microbial taxa that may be potentially tolerant to elevated levels of heavy metals and hydrocarbons. For the 16S rRNA marker, nine bacterial taxa including *Iamia*, *Paenibacillus*, *Alteromonas*, and *Blastococcus* were enriched in contaminated soils compared to uncontaminated soils (LEfSe LDA > 3, *p* < 0.05; Fig. 5A). Furthermore, *Actinochloris* and Colpodida were strongly associated with contaminated soils for 18S rRNA microbiome profiles (LEfSe LDA > 3, *p* < 0.05; Fig. 5B). Three fungal groups (*Alternaria*, *Furcasterigmium*, and *Stachybotrys*) were significantly more abundant in contaminated than uncontaminated soils (LEfSe LDA > 4, *p* < 0.05; Fig. 5C).

## Discussion

### Principal Findings of Study

We used eDNA metabarcoding to assess the soil microbiome dynamics in response to long-term contamination at a former rail yard and urban brownfield site. We quantified contamination levels by measuring soil concentrations of heavy metals, petroleum hydrocarbons, and polycyclic aromatic hydrocarbons, and related their concentrations to bacterial, eukaryotic, and fungal community alpha and beta diversity. The data showed that soil eukaryotic and fungal richness increased at higher concentrations of lead and cobalt, respectively. Furthermore, soil microbiomes were partitioned by depth but did not differ between contaminated and uncontaminated surface soils. Concentrations of cobalt, chromium, and lead partially explained the variation in bacterial and eukaryotic community structure, while fungal communities were more sensitive to arsenic and cobalt. 16S rRNA bacterial communities were also significantly correlated with arsenic levels. Lastly, we identified several microbial groups that were enriched in contaminated surface soils relative to uncontaminated soils for all three markers studied. Among these groups were the bacterial genera *Paenibacillus*, *Alteromonas*, and *Iamia*, the eukaryotic genus *Actinochloris*, and the fungal genus *Alternaria*. Collectively, our findings show that heavy metals are significantly correlated with soil microbiome composition and diversity at this urban brownfield site. Several microbial groups are potentially tolerant to heavy metals and future studies should determine their functional capacity to metabolize, excrete, or bioaccumulate the heavy metals found in brownfield soils.

### Brownfield Site Soil Microbiome Composition Resembles That of Other Brownfield Sites

Broadly, the study site’s soil microbiome profiles resemble those of surface soils polluted with heavy metals in other urban railyards [8], Pb/Zn smelters [50], contaminated forest soils [51], and coastal aquaculture areas [52]. As was observed in this study, Proteobacteria, Acidobacteria, and Actinobacteria were also the most dominant bacterial phyla found in chromium-contaminated soils in the Yunnan Province (China) [53]. Our brownfield site mirrored former steel industry soils from northeastern France, which reported Ascomycota as the dominant fungal phylum [28]. Furthermore, similar to our study Alphaproteobacteria and Deltaproteobacteria were abundant bacterial classes in the soils of an urban brownfield rail yard located in Jersey City, NJ [8, 54]. This same site also noted Sordariomycetes and Eurotiomycetes as abundant fungal classes. Our findings are consistent with a prior study that detected the eukaryotic phyla Basidiomycota and Streptophyta (land plants and green algae) at high abundances in a silver-lead–zinc mine from New South Wales, Australia [55]. In conclusion, the microbial communities at the “Bowtie” brownfield site are similar to other heavy-metal contaminated sites from varying geographic regions.

### Hazardous Chemicals Are Associated with Microbiome Alpha and Beta Diversity

In our study, heavy-metal concentrations were associated with microbiome alpha and beta diversity. The richness of 18S rRNA profiles increased with greater concentrations of lead while the richness of fungal communities increased with high cobalt concentrations.

In our study, the soil community has been exposed to heavy metals for over 30 years, potentially allowing the microbial communities to develop resistance or tolerance. This could give rise to a diverse soil community over time. Resistance mechanisms can take a variety of forms in organisms, including the expulsion of heavy metals to outside of the cell via efflux pumps, transformation of toxic metals into less toxic forms, absorption into the cell wall, or sequestration into subcellular compartments [18, 20]. Without these resistance mechanisms, heavy metals can damage cells and interfere with chromosome replication, DNA synthesis, protein synthesis, nucleic acid metabolism, cellular respiration, and the citric acid cycle, or lead to oxidative stress and enzyme inhibition [56, 57].

Several genera of filamentous fungi (*Trichoderma*, *Penicillium*, *Paecilomyces*, *Pythium*, *Rhizopus*, *Mortierella*, and *Aspergillus*) belonging to orders of fungi recovered from this brownfield site (e.g., Eurotiales, Hypocreales) show bioabsorption of cobalt in serpentine soils from the Andaman Islands, India, which contain high levels of heavy metals [58]. Other studies show that vacuolar sequestration of heavy metals is a common detoxification mechanism in eukaryotes, particularly in yeast cells [59]. Thus, it appears that mechanisms for cobalt and lead tolerance are found in fungi and other eukaryotes.

In our study, concentrations of cobalt, chromium, and lead partially predicted bacterial and eukaryotic community structure, whereas fungal communities varied with arsenic and cobalt concentrations. A plethora of studies have detected associations between heavy-metal levels and soil microbiome structure in pastures, phytoremediation experimental plots, government transportation dump sites, agricultural soils, and heavy-metal experimental plots [60–64]. A previous brownfield railyard similarly reported that bacterial community structure was correlated with lead, in addition to copper and zinc but found that none of the heavy metals were significantly associated with fungal community structure [8].

In our study, both bacterial and fungal communities covaried with arsenic concentrations. These findings are unexpected since genes that can detoxify or metabolize arsenic (ArsB, ArsM, AioAB) are widespread among bacteria [65], so we would have expected a weak correlation with arsenic concentrations. Arsenic is typically present as arsenite (As(III)) in soils, and bacteria can pump out As(III) via an efflux pump, or reduce less common forms of arsenic to As(III) [66]. Furthermore, another study showed that the most abundant metal-resistance genes in soils from an abandoned copper mine were associated with Cu, As, and Fe resistance [67]. Nonetheless, a silver-lead–zinc mine also reported the soil bacterial community to vary with As concentrations [55]. Thus, the relationship between bacterial community structure and heavy-metal concentrations likely depends on soil properties like pH, mineral content, and moisture. For fungi, arsenic resistance is more limited than in bacteria and has mostly been observed in arbuscular mycorrhizal fungi [68]. These fungi can reduce As(V) to As(III) and pump As(III) out of cells. However, at our study site arbuscular mycorrhiza-forming fungi were absent, which is perhaps why fungal communities were shaped by arsenic levels.

Both bacterial and eukaryotic communities were significantly associated with lead concentrations, while fungal communities were not. Similarly, in a heavy-metal contaminated reservoir in China, the relative abundances of bacterial groups were associated with Cr, Pb, and Zn [69]. In a lead smelter that recycles lead acid batteries, bacterial communities were primarily shaped by soil type and secondarily by Pb content [70]. Lead lacks a significant biological function, is regarded as toxic for nearly all microorganisms [71], and has been shown to reduce microbial biomass and metabolic activity via enzyme inactivation [72]. At very low Pb levels (< 10 mg/kg) however, microbial development can occur [73]. Thus, different groups of bacteria could survive at high or low Pb levels. Plants can accumulate lead as well if associated with rhizospheric bacteria or mycorrhizal fungi [74, 75]. Fungi on the contrary are a versatile biosorption group [76, 77] and have the ability to potentially bind and bioaccumulate lead, which may have contributed to their general tolerance to Pb at the “Bowtie” brownfield site.

### Microbial Taxa with the Potential for Heavy-Metal Bioremediation

LEfSe analyses identified several microbial taxa that were enriched in contaminated surface soils (Fig. 5) compared to uncontaminated surface soils. These microbial taxa are tolerant of heavy metals and may contain the metabolic machinery required for soil bioremediation. Identified groups came from the bacterial genera *Iamia*, *Paenibacillus*, and *Alteromonas*. In active nonferrous mine tailings in China and in areas of mining and ore smelting, *Iamia* is positively correlated with the heavy metals Zn, Sb, Pb, As, and Cd [78]. This genus however remains poorly understood because few species have been isolated and additional research is required to understand their potential for heavy-metal resistance and tolerance. Bacterial members from the genus *Paenibacillus* [79] possess metal-resistant genes as an adaptation to metal pollution [80], and several strains have the capacity to bioadsorb heavy metals (Zn, Cd, Cr, Co, and Pb) from industrially polluted soils [81]. They can also reduce arsenic to less toxic forms [82]. *Alteromonas* sp. from marine environments can uptake mercury and two species from the Red Sea can remove a range of heavy metals from aqueous solution [83–85].

Two eukaryotic genera, *Actinochloris* and Colpodida, were also enriched in contaminated surface soils and may possess genes which confer multiple metal resistance [86]. They may do this by accumulating metals in their cell walls and secreting them via extracellular polymeric substances [87–90]. *Actinochloris* is a genus of green algae that forms part of the soil phycoflora [91] in aerotechnogenically polluted spruce forest soils [92] and high-salinity biocrusts [93]. Their potential for bioremediation has not yet been explicitly examined. Colpodida is an order of bacterivorous ciliates [94] and contains certain species that can be used as a bioassay for heavy metals in sewage sludge [95]. They can also enhance the in vitro microbial degradation of crude petroleum [96].

Lastly, at the study site three fungal genera, *Alternaria*, *Furcasterigmium*, and *Stachybotrys*, were more abundant in contaminated soils than uncontaminated soils. These three groups have the ability to leach, absorb, and transform heavy metals [97]. Filamentous fungi can accumulate heavy metals and metalloids [98, 99] and the genus *Alternaria* specifically can tolerate a range of heavy metals including copper, lead, silver, and mercury [26, 100]. This group of fungi can degrade PAH in vitro [26]. On the contrary, much less is known about the fungal genus *Furcasterigmium*; it is a fungal group commonly found in drinking water storage tanks and within wet walls [101]. *Stachybotrys* are filamentous fungi common in soil, decaying plant material, and submerged wood in mangroves [102]. One species of this genus, *S*. *chartarum* can absorb copper ions but other studies show that their growth can be affected by high levels of cobalt and copper [103]. Thus, the organic or inorganic pollutant degrading capacities of *Stachybotrys* are inconclusive.

### Limitations

Soil communities are strongly shaped by a variety of soil parameters, including soil pH, moisture, compactness, salinity, acidity, and alkalinity, as well as by nutrient content, including organic carbon, nitrogen, oxygen, phosphorus, sodium, and potassium [104–106]. In our study, we did not quantify any of these soil parameters, beyond concentrations of heavy metals and polycyclic hydrocarbons, which limits our conclusions. Our results would be more comprehensive if measurements of soil characteristics had been taken. Nonetheless, even with these missing parameters, our findings enhance the body of literature and shed light on the microbial ecology of this brownfield site which was previously unexplored. Another limitation of our study involved our measurements of heavy metals; the measurements were not very granular, detection was low, and future studies will need to assess each heavy metal individually [107] across a diversity of depths. This will be critical for detecting pollution of heavy metals at and below the surface; of heavy metals that may have leached to deeper layers in the soil, which may have occurred at our site. Heavy metals will also need to be observed in tandem as the bioavailability, and hence, toxicity of a heavy metal is dependent on its interactions with other elements (e.g., chromium with manganese and iron forms a less biologically active species) [107]. Thus, we recommend future studies consider soil parameters and assess heavy metals holistically.

Additionally, our findings, particularly from the fungal data should be interpreted with caution, as the majority of reads for over half of the samples were unclassified and these samples were excluded from statistical analyses. All extant ITS primers are designed based on databases of available sequences; however, there are still large gaps in fungal databases, as they poorly represent the estimated 6.2 million species [108]. Taxonomic resolution was also low for the 18S rRNA marker data, since over 50% of sequences in any given sample could not be classified beyond kingdom level (Eukaryota). For these reasons, we highly recommend that future studies employ metagenomic surveys to assay microbial community structure and function [109]. Metagenomics sequencing is a promising avenue to further research on soil microbiome tolerance and resistance to heavy metals, particularly in soil environments that have not been studied before.

### Implications in Restoration Ecology of Urban Brownfields

Urban brownfield restoration and rehabilitation plays a significant role in reducing urban sprawl [110, 111], boosting the economy [112], and improving quality of life in underserved neighborhoods [113]. Additional ecological benefits include carbon sequestration, flood control, and climate regulation [114]. However, in order to develop strategies to achieve this restored state, we need to understand the unique ecological community and environmental characteristics of the contaminated site [8]. Sustainable alternatives for restoration include phyto- and bioremediation, which combine the ability of plants, microbes, and fungi to accumulate, transform, translocate, and biodegrade contaminants onsite for land decontamination [115]. Therefore, identification of candidate native microbial species is a crucial component in optimizing the removal of the specific contaminants at brownfields. Once identified, native microorganisms including plant-associated mycorrhizae [116] and compost enhanced with natural amendments can be applied [117]. The use of next-generation sequencing can be used to analyze the treated soil overtime to provide insight into the progress and effectiveness of the treatment. Future studies can use metagenomics and metatranscriptomics to provide the simultaneous results of community composition and gene expression [118] to better understand the metabolic capacity of microorganisms to bioremediate heavy-metal pollutants at brownfield sites.

## Supplementary Information

Below is the link to the electronic supplementary material.

Supplementary file1 (PDF 1053 KB)

Supplementary file2 (XLSX 218 KB)

## Data Availability

All sequence data are publicly available on the NCBI Sequence Read Archive (SRA) under BioProject PRJNA742242 and accession numbers SAMN19946165–SAMN19946227. R code and scripts for analyzing all data and building the figures shown in this article are available on GitHub (https://github.com/rojascon/Soil_microbiome_contaminanted_brownfield_site_.git).
