# Supplementary material for: Soil Microbial Community Composition and Tolerance to Contaminants in an Urban Brownfield Site
Source: Microb Ecol. 2022 Jul 8;85(3):998–1012. doi: 10.1007/s00248-022-02061-1 (PMC10156844; doi:10.1007/s00248-022-02061-1)

SUPPLEMENTARY TEXT AND FIGURES FOR:

Soil microbial community composition and tolerance to contaminants in an urban brownfield site

Maura Palacios Mejia*[1†], Connie A. Rojas[2†], Emily Curd[3], Mark A. Renshaw[4], Kiumars Edalati[1], Beverly Shih[1], Nitin Vincent[1], Meixi Lin[1], Peggy H. Nguyen[5], Robert Wayne[1], Kelsey Jessup[6], Sophie S. Parker[6]

[1] Ecology & Evolutionary Biology, University of California, Los Angeles, Los Angeles, CA, USA
[2] Ecology, Evolution, and Behavior Program, Michigan State University, Lansing, MI, USA
[3] Natural Science, Landmark College, Putney, VT, USA
[4] Cherokee Federal, USGS Wetland and Aquatic Research Center, Gainesville, FL, USA
[5] Institute of the Environment and Sustainability, University of California, Los Angeles, Los Angeles, CA, USA
[6] The Nature Conservancy, Los Angeles, CA, USA

† denotes co-first authors

Name and email address of corresponding author:
Maura Palacios Mejia, mepalacios@ucla.edu

SUPPLEMENTARY TEXT

Materials and Methods

Study site and sample collection.

At the 12 sites, surface samples (0.1524 m) were collected by hand using disposable scoops, and subsurface samples (1.524–6.096 m) were collected using a hardened steel-core barrel lined with an acetate sleeve, driven every 1.524 meters (5 ft) below ground by a direct-push drill rig. The samples were collected according to EPA Method 5035A using sample-dedicated Terra Core kits (En Novative), by pushing the “T” bar into an undisturbed portion of the soil from the acetate sleeve. Four Terra Core sample containers were filled at each sample site and placed into a resealable bag. All samples were immediately labeled and chilled to 4 °C by placing them on ice in an insulated cooler. Then, they were hand-shipped under chain-of-custody control to the laboratory for chemical analysis.

Sample processing: environmental parameters & eDNA metabarcoding library preparation

Two rounds of polymerase chain reaction (PCR) were used to amplify three targeted metabarcodes from soil samples for microorganisms from the Earth Microbiome Project: 16S rRNA, 18S rRNA, and fungal ITS (FITS). Primers for each of the three assays were ordered from Integrated DNA Technologies (Iowa) as originally published. Additionally, Illumina Nextera Transposase Adapters were included on the 5′ end of the synthesized primer pairs: TCGTCGGCAGCGTCAGATGTGTATAAGAGACAG (forward) and GTCTCGTGGGCTCGGAGATGTGTATAAGAGACAG (reverse). The 20 µL PCR mixes contained the following: 4 µL of 5X GoTaq Flexi Buffer (Promega), 0.4 µL of 10 mM dNTPs, 1.6 µL of 25 mM $MgCl_2$, 1 µL of 10 µM forward primer, 1 µL of 10 µM reverse primer, 8 µg of Bovine Serum Albumin (BSA, 20 mg/ml, VWR, Pennsylvania), 0.15 µL of GoTaq G2 Flexi

DNA Polymerase (Promega), 4 µL of DNA extract, and 5.85 µL of sterile water. Cycling protocols for each assay began with a 3minute denaturing period at 95°C and ended with a single 10-minute extension step at 72°C. The three step cycling involved denaturation at 95°C for 30 seconds, annealing for 45 seconds (temperatures and cycle numbers given in Table S2), and elongation at 72°C for 60 seconds. PCR products from the 16S rRNA, 18S rRNA, and FITS primers were pooled into one mix in equal volumes. They were cleaned with MagBind® TotalPure NGS (Omega BioTek Inc) magnetic beads at a ratio of 1 (beads):1 (DNA) and following the manufacturer's recommendations to remove fragments shorter than 350 bp.

During the second round of PCR, we added sample-specific dual indexes and used the PCR products described above as templates. Each pooled PCR amplicon mix was used as a template for a different sample; consequently, one library was generated for each eDNA sample. The second round PCR mix (30 µL) consisted of the following: 6 µL of 5X GoTaq Flexi Buffer (Promega), 0.6 µL of 10 mM dNTPs, 2.4 µL of 25 mM $MgCl_2$, 1.5 µL of 10 µM forward primer, 1.5 µL of 10 µM reverse primer, 0.15 µL of GoTaq G2 Flexi DNA Polymerase (Promega), 3 µL of the pooled PCR amplicon mix, and 14.85 µL of sterile water. The forward and reverse primers, which included the remaining Illumina adaptor sequence, were: AATGATACGGCGACCACCGAGATCTACAC[i5]TCGTCGGCAGCGTC (forward) and CAAGCAGAAGACGGCATACGAGAT[i7]GTCTCGTGGGCTCGG (reverse), and Nextera DNA indexes (i5 and i7) for dual indexing (Illumina, 2020). PCR products were cleaned with Mag Bind® TotalPure NGS (Omega Bio Tek Inc) magnetic beads at a ratio of 0.8 (beads):1 (DNA), following the manufacturer's recommendations to remove fragments shorter than 300 bp.

Identifying collinear environmental parameters

Of the hydrocarbons and heavy metals assessed and quantified (Table S2), only a fraction were considered for statistical analyses (Table S3). Those that did not display sufficient variation between samples (e.g. all samples had the same concentration) were excluded. Furthermore, hydrocarbons and heavy metals that were highly correlated with each other were also not included. For this, we constructed a correlation matrix of environmental predictors in R and identified those that were strongly correlated with one another ($r > 0.5$) and statistically significant ($\alpha = 0.01$) (Table S3). These were excluded from statistical analyses, leaving arsenic, cobalt, chromium, lead, and benzo(a)pyrene to be included in our statistical models (Table S3).

Samples from the surface (0.1524 m) were classified as “contaminated” if their values exceeded regional environmental screening levels and guidelines set by the respective overseeing agency for one or more of the following: heavy metals, TPHs, and/or PAHs (Table S2). Agencies included the Environmental Protection Agency [5], California Department of Toxic Substances Control (DTSC, 2019), and the San Francisco Regional Water Quality Control Boards (California San Francisco Bay RWQCB, 2019) for residential soils. Samples from the surface were classified as “uncontaminated” if that was not the case.

Results

Levels of contamination in soil of an urban brownfield site

Of the 17 heavy metals, 2 petroleum hydrocarbons, and 16 polycyclic aromatic hydrocarbons we quantified, only two metals, one petroleum hydrocarbon, and four aromatic hydrocarbons exceeded the regional screening levels set by the EPA, California DTSC, and San Francisco RWQCB for residential soils. All exceedances were found in surface samples. Specifically, the arsenic levels of six surface samples (range: 0–0.43 mg/kg) exceeded regional screening levels established by the EPA (0.68 mg/kg; Table S2) and DTSC (0.11 mg/kg; Table S2). The lead concentrations of two surface samples (1.6–140 mg/kg) surpassed the DTSC-established level of 80 mg/kg. TPH-d levels of four surface soil samples (320–640 mg/kg) exceeded the preferred ESL residential soil concentrations of 260 mg/kg (Table S2). For PAHs, two samples (750-4,000 µg/kg) had concentrations higher than the EPA RSL for rbenzo[a]pyrene (110 µg/kg), one sample (140,000 µg/kg) exceeded EPA RSLs for benzo[b]fluoranthene levels (1,100 µg/kg), and three samples (4,600-46,000 ug/kg) exceeded EPA-RSLs for benzo[k]fluoranthene (11,00 µg/kg; Table S2). Three soil samples (4,400-62,000 ug/kg) contained indeno (1,2,3-cd) pyrene concentrations exceeding EPA RSL guidelines (Table S2).

SUPPLEMENTARY FIGURES

Fig S1 - rarefaction curves
Fig S2 - barplot of microbiome phylum abundances for each marker
Fig S3 - Chao2 richness plots
Fig S4 - PCoA ordinations with samples color-coded by depth

Figure S1. Rarefaction curves of soil microbiome ASV richness for the three gene markers studied. Plotted are the number of ASVs (ASV Richness) recovered with an increasing number of sequences, after subsampling to 45,000 sequences/sample for 16S rRNA profiles, 20,000 sequences/sample for 18S rRNA profiles, and 370 sequences/sample for FITS profiles. Samples with fewer sequences than the cutoff were excluded. Each curve represents a unique sample and is color-coded by sample depth. All curves plateau indicating that the sequencing cutoff was appropriate and accurately captured community diversity.

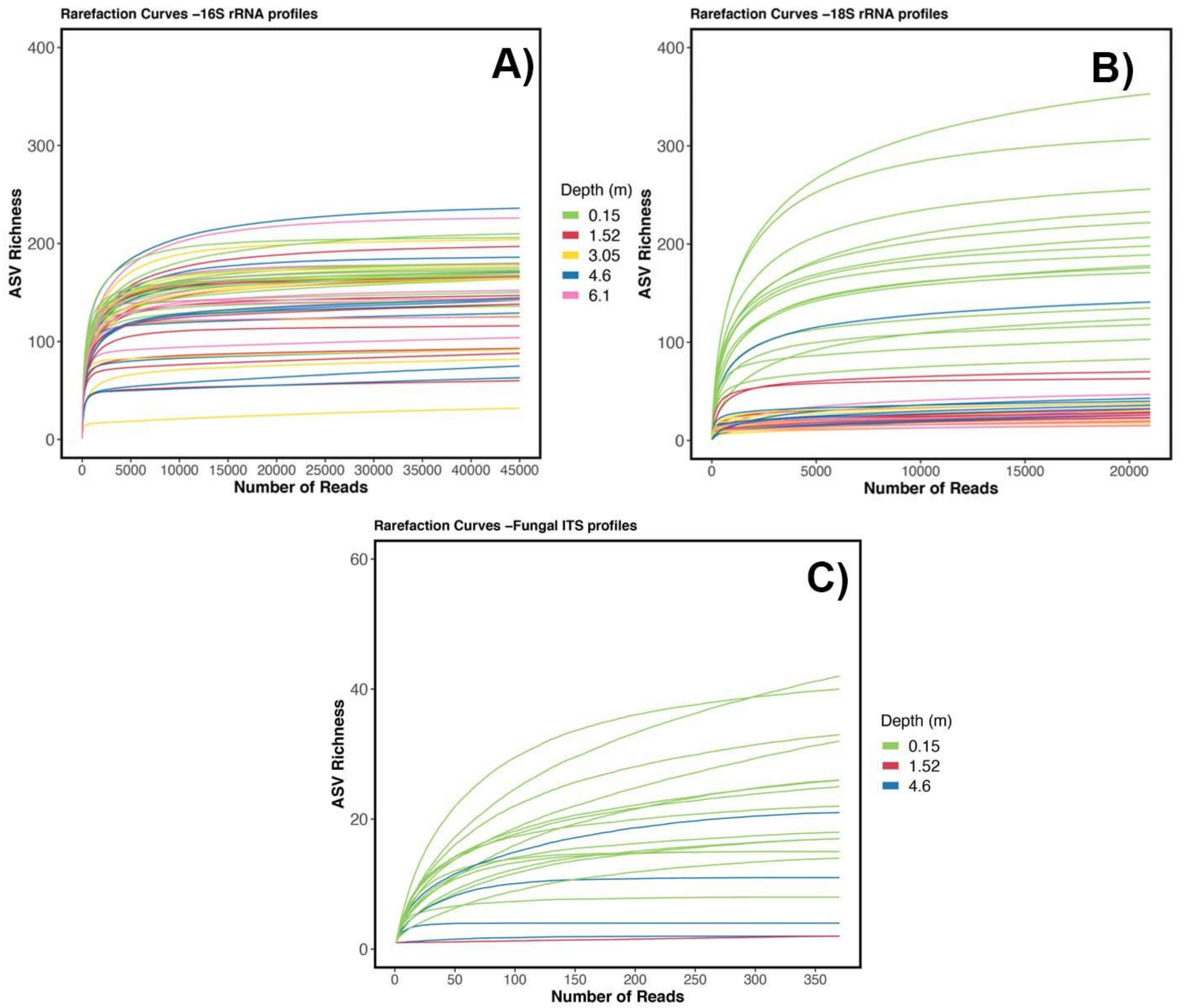

Figure S2. Predominant phyla of the soil microbiome in a contaminated brownfield site. Stacked bar plots showing the relative frequency of sequences assigned to each microbial phylum across samples for **A)**16S rRNA, **B)** 18S rRNA, and **C)** fungal ITS gene profiles. Samples are grouped by depth, and each color represents a microbial phylum. Microbial abundance data was rarefied for these plots (42,000 reads - 16S rRNA data, 21,000 reads - 18S rRNA data, and 370 reads per sample - FITS data).

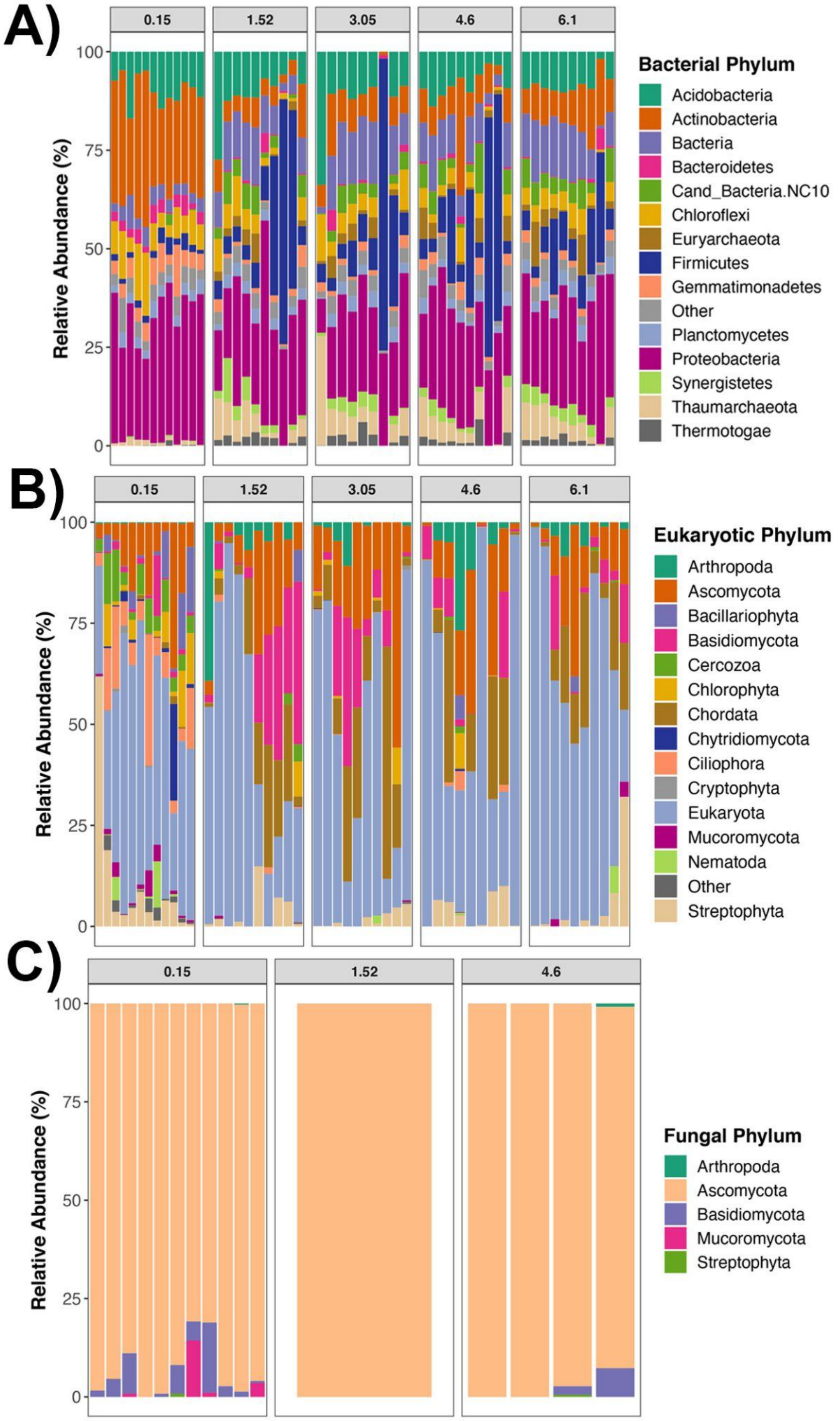

Figure S3. Soil microbiome richness is correlated with concentrations of heavy metals. Plots showing Chao 2 richness predicted from a linear model relating the concentrations of Lead (μg/kg) and Cobalt (μg/kg) to soil microbiome alpha diversity for A) 18S rRNA profiles and B) fungal ITS profiles. Only samples from depths with known concentrations of heavy metals were included. The shaded areas represent 95% CI. Model statistical output is in Table S5.

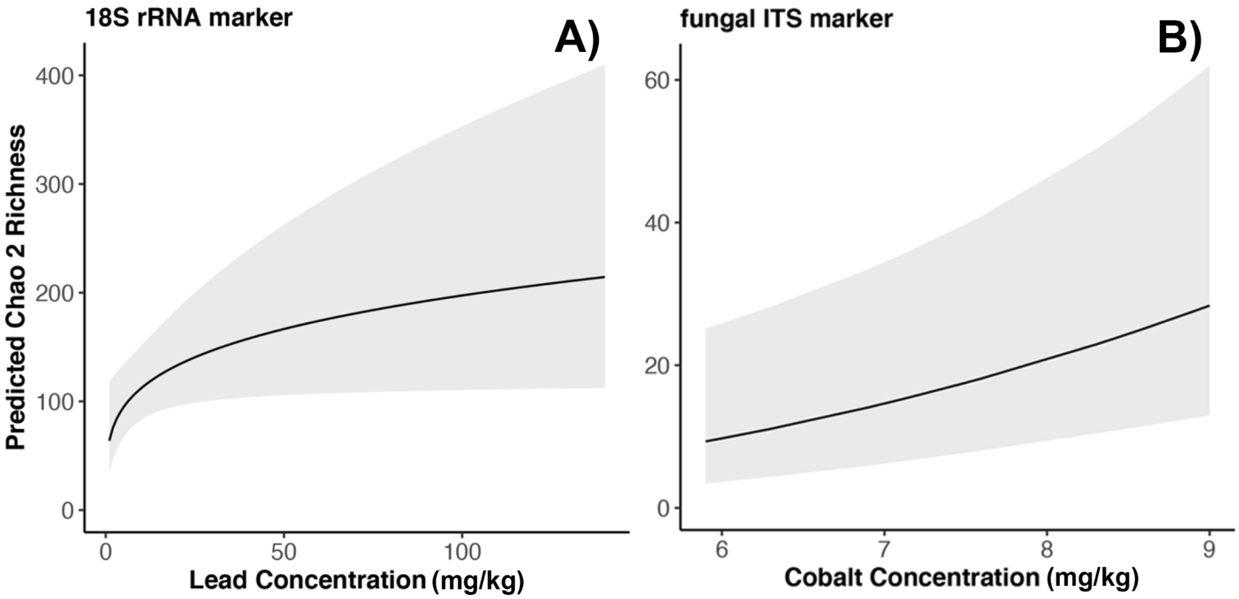

Figure S4. Stratification of soil microbiome by sampling depth. PCoA plots constructed from Jaccard dissimilarity matrices for A) 16S rRNA, B) 18S rRNA, and C) fungal ITS data. Each point represents a sample and is color-coded by depth (left) or a binary category for pollutant contamination (surface samples only; right). Closeness of points indicates high community similarity. The percentage of variance accounted for by each PC axis is shown in the axis labels.

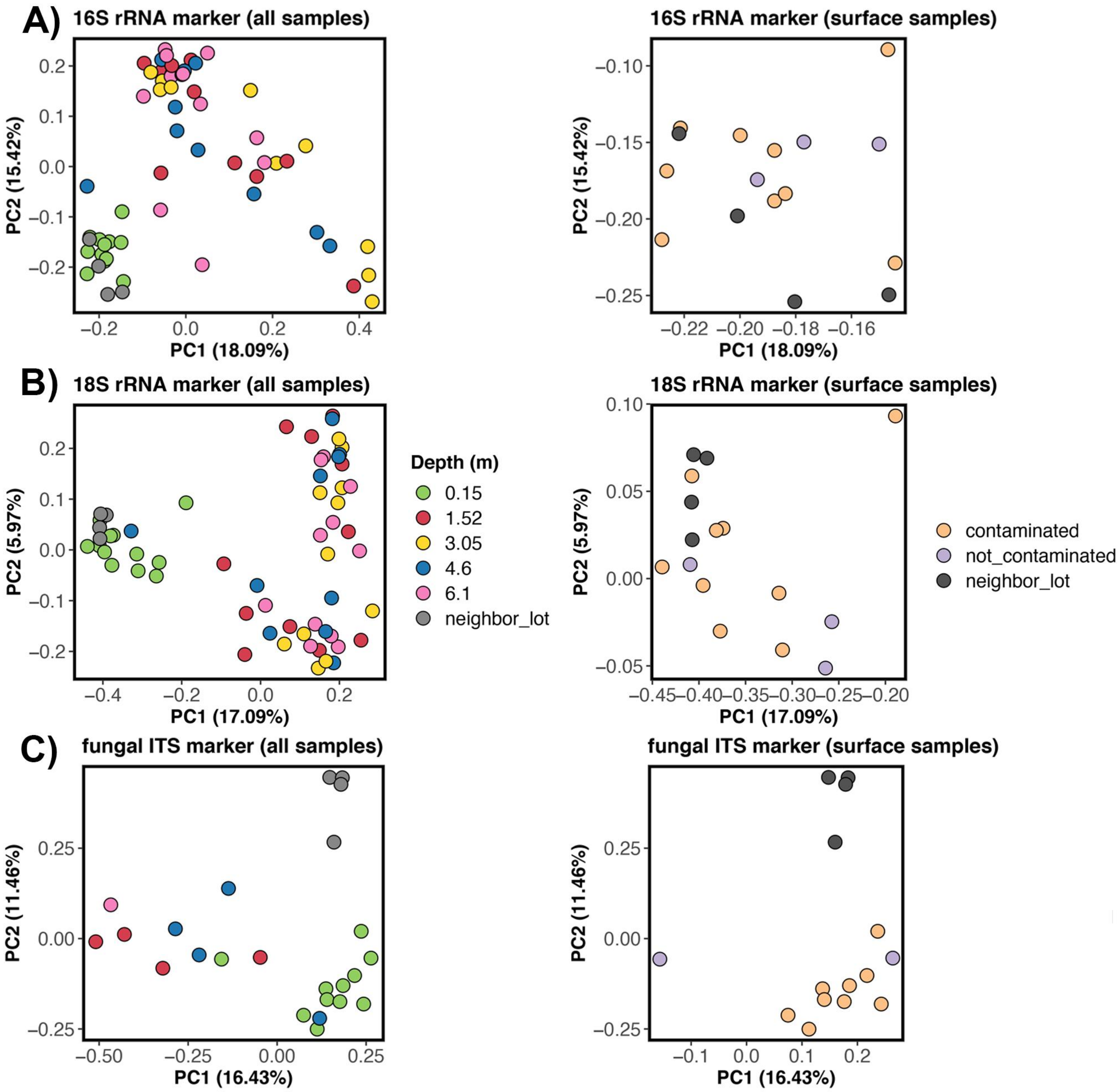

Supplement: Supplementary file 1 — Supplementary file1 (PDF 1053 KB) [file 248_2022_2061_MOESM1_ESM.pdf]
